# The Influence of Sexual Orientation on the Perceived Fit of Male Applicants for Both Male- and Female-Typed Jobs

**DOI:** 10.3389/fpsyg.2018.00656

**Published:** 2018-05-03

**Authors:** Heather M. Clarke, Kara A. Arnold

**Affiliations:** ^1^Austin E. Cofrin School of Business, University of Wisconsin, Green Bay, WI, United States; ^2^Faculty of Business Administration, Memorial University of Newfoundland, St. John's, NL, Canada

**Keywords:** gender stereotypes, sexual orientation, gender-typed work, implicit inversion, lack of fit, role congruity, gay men, heterosexual men

## Abstract

Research demonstrates the bias faced by individuals engaged in occupations that are perceived as inconsistent with their gender. The lack of fit model and role congruity theory explain how gender stereotypes give rise to the perception that an individual lacks the attributes necessary to be successful in a gender-incongruent job. Men employed in jobs traditionally held by women are perceived as wimpy and undeserving of respect. The majority of studies in this area have, however, failed to account for the sexual orientation of the individual being rated. Therefore, we carried out an experiment where 128 adults with experience in recruitment and selection, recruited through Qualtrics, rated heterosexual and gay male applicants applying for a gender-typed job. The heterosexual male was rated less effectual, less respect-worthy, and less hirable in the female-typed job condition than in the male-typed job condition. The gay male applicant, however, was rated similarly on all criteria across job gender-types, suggesting the gay male applicant was viewed as androgynous rather than high in femininity and low in masculinity as inferred by implicit inversion theory. The implications of these findings are discussed.

## Introduction

Research has evidenced employment discrimination faced by gay men (Horvath and Ryan, 2003; Drydakis, 2015). The issue of prejudice against gay men is made all the more poignant with the current administration in the United States openly opposing gay rights; explicitly urging the courts to find that the Title VII protections against employment discrimination do not prohibit discrimination based on sexual orientation (Barbash, 2017). In an interview with the New Yorker, President Trump joked that Vice President Pence “wants to hang all gays” (Amatulli, 2017). Such actions may be viewed as legitimizing bias against members of the LGBTQ+ community and creating a climate in which employment discrimination against members of that community becomes normative.

Studies have also demonstrated that bias or prejudice manifests within the context of gender-typed work. For instance, negative appraisals result when an individual engages in an occupation that is perceived to be incongruent with the traits and attributes associated with that individual's gender (Heilman, 1983; Eagly and Karau, 2002; Heilman et al., 2004; Heilman and Wallen, 2010). Women employed in male-typed jobs are assumed to be less competent than their male counterparts (Heilman et al., 2004). Men employed in female-typed jobs are viewed as ineffectual and undeserving of respect (Heilman and Wallen, 2010).

Past studies have demonstrated that gender stereotypes of gay men tend to be in the opposite direction of those about heterosexual men (e.g., Blashill and Powlishta, 2009). According to implicit inversion theory, gay men are perceived to be highly feminine and low in masculinity (Kite and Deaux, 1987). This inversion of gender stereotypes has been largely unaccounted for in studies on gender-typed employment and it suggests that sexual orientation should have an effect on perceptions of individuals engaged in gender-typed work. The purpose of the current study was to examine perceptions of male applicants for gender-typed jobs. Specifically, we investigate how sexual orientation interacts with the gender-type of the job to influencing the perceived suitability of job applicants.

The literature on gender-typed jobs has focused primarily on heterosexuals, however gay men have greater interest in gender-atypical careers than heterosexual men (Ellis et al., 2012; Ueno et al., 2013). It is therefore important to examine how gay men are perceived as applicants for gender-typed jobs. Finally, much of the research on evaluations of targets in gender-typed jobs has employed within-person designs, which are susceptible to demand effects, and employ undergraduate student participants. Our final objective is to determine whether previous findings are replicated with a more conservative, between-person design, and with participants that are employed adults with experience in recruitment and selection.

### Sexual orientation and gender stereotypes

Gender stereotypes are “categorical beliefs regarding the traits and behavioral characteristics ascribed to individuals on the basis of their gender” (Duehr and Bono, 2006, p. 816). Gender stereotypes lead us to attribute certain physical characteristics, personality traits, and behaviors to women and others to men (Heilman, 1983; Eagly, 1987). The stereotype of women is that of being communal; i.e., possessing traits like kind, caring, and nurturing, and as engaging in activities (e.g., sewing) and occupations (e.g., nursing) traditionally associated with women (Heilman, 1983; Eagly, 1987). Men are stereotyped as being strong, competent, and decisive, as engaging in normatively male behaviors (e.g., riding a motorcycle) and occupations (e.g., manager) (Heilman, 1983; Eagly, 1987).

Notwithstanding that masculinity and femininity are orthogonal constructs (Bem, 1974; Spence et al., 1974), when making judgments about others, we employ a bipolar model of gender that places masculinity and femininity at opposite ends of the same continuum (Foushee et al., 1979; Biernat, 1991). Under this bipolar model, masculinity and femininity are inversely related such that women are assumed to be both high in femininity and low in masculinity, while men are assumed to be high in masculinity and low in femininity. These stereotypes, however, apply only to heterosexual individuals.

Implicit inversion theory states that gay men and lesbians are viewed as more similar to cross-sex heterosexuals than to same-sex heterosexuals (Deaux and Kite, 1985; Kite and Deaux, 1987; Blashill and Powlishta, 2009). That is, gay men are viewed as less masculine and more feminine than heterosexual men, and lesbians are viewed as more masculine and less feminine than heterosexual women. Empirical investigations have largely found support for implicit inversion theory (Taylor, 1983; Page and Yee, 1985; Kite and Deaux, 1987; Jackson and Sullivan, 1990; Madon, 1997; Wong et al., 1999; Blashill and Powlishta, 2009). This inversion of gender stereotypes should influence the perceived suitability of applicants for gender-typed jobs.

### Sexual orientation and gender-typed work

Not all work is gender-typed, but jobs that have traditionally been held exclusively or almost exclusively by one gender come to be viewed as better suited for that gender. The assumption is that to be successful in that job, one must possess the traits attributed to its gender-type. Occupations like engineer or construction worker are considered to be male-typed jobs and are believed to require male, agentic characteristics (Heilman, 1983; Eagly, 1987; Eagly and Karau, 2002). Whereas jobs like child-care worker or nurse are female-typed.

One theory developed to explain the bias or prejudice experienced by individuals working in gender-inconsistent occupations is the lack of fit model (Heilman, 1983). The lack of fit model explains how gender stereotypes interact with beliefs about the gender-type of work to produce judgments or evaluations about performance (Heilman, 1983). Expectations about how successful or unsuccessful a person will be in a job are determined by the perceived fit of that person's attributes and the traits and abilities believed to be necessary to perform the job. These expectations in turn influence how performance is evaluated and rewarded (Heilman, 1983). Similarly, role congruity theory predicts that prejudice can arise when stereotypic traits of a particular group are incongruent with the attributes believed to be necessary to be successful in a particular role (Eagly and Karau, 2002).

Studies on evaluations of individuals in gender-inconsistent jobs that have found support for these theories have largely investigated perceptions of women employed in male-typed occupations, and found that women employed in male-typed jobs are assumed to be less competent than their male counterparts (Heilman et al., 2004). Further, when a woman proves to be competent in her male-typed role, she is rated competent but also unlikeable and interpersonally hostile. Heilman and Wallen (2010) investigated perceptions of men in gender-inconsistent occupational roles, and they found that a male employed in a female-typed job was rated more ineffectual and less deserving of respect than a male employed in a male-typed job. This is because men engaged in roles traditionally occupied by women leads to the assumption that those men must lack the masculine, agentic traits that we expect of men. Other research similarly suggests that men's gender-inconsistent behavior may result in them being viewed as wimpy and undeserving of respect (Rudman, 1998; Rudman and Glick, 1999). The majority of this past research, however, has not investigated the role of sexual orientation. Given the likelihood of a heteronormative assumption, it is probable that participants in previous research have assumed the targets being rated to be heterosexual.

For a heterosexual male, the mere knowledge that he is employed in or applying for a female-typed job should lead to decreased ratings of respect and effectuality. Despite the fact that prior research examined the backlash experienced by successful males in female-typed jobs (Heilman and Wallen, 2010), information about successful performance should not be necessary to evoke social punishment for heterosexual males, because female-typed work is not valued or viewed with as much respect as male-typed work (England, 2010; Torre, 2014). It is also more acceptable for a woman to hold a male-typed job than a man to hold a female-typed job (Didonato and Strough, 2013), because female-typed jobs tend to be lower in status and pay. Thus, the simple fact that a heterosexual male is employed in or applying for a female-typed job will give rise to the assumption that he must be ineffectual and undeserving of respect.

Further, the knowledge that a man is applying for a female-typed job leads to the assumption that he possesses the communal, expressive, warm feminine traits necessary to perform that job. This simultaneously leads to the assumption that he lacks prescribed masculine agentic traits (Biernat, 1991), giving rise to the judgment of him as being undeserving of respect and ineffectual. Heterosexual males will therefore be viewed as less effectual and less deserving of respect when applying for a female-typed job than when applying for a male-typed job, and will therefore be less likely to be hired for a female-typed job than for a male-typed job.

It has become more common for both males and females to enter occupations that have been traditionally viewed as appropriate for the opposite gender (Whittock, 2002; Watts, 2009). Notwithstanding that gay men display greater preference for female-typed work than do heterosexual men (Ellis et al., 2012; Ueno et al., 2013), little research has accounted for sexual orientation within the context of gender-typed work, and those studies that have, appear to provide inconsistent results. A recent study demonstrated that gay applicants were less likely than heterosexual male applicants to be invited for interviews for male-typed job (Drydakis, 2015). In contrast, Niedlich and Steffens (2015) found that gay men applying for leadership positions were viewed as possessing equal levels of feminine and masculine traits.

If gay males are perceived as possessing feminine attributes, traditional gender norms may not apply to them. It is therefore possible that gay males are not seen as less respect-worthy or more ineffectual when they apply for female-typed jobs. One aspect of gay prejudice is in fact the perception that gay men violate traditional gender roles by being high in femininity and low in masculinity (Levahot and Lambert, 2007). Therefore, the gender-type of the job should have no bearing on the ineffectuality or respect ratings of gay male applicants.

However, because gay men are presumed to be high in femininity and low in masculinity (Blashill and Powlishta, 2009), they will likely be perceived as a better fit for female-typed work than male-typed work and therefore be more likely to be hired for female-typed jobs. In fact, some jobs that have traditionally been viewed as female-typed jobs are also viewed as “gay” jobs because it is common to find gay men performing successfully in those occupations (see, e.g., Anteby and Anderson, 2014). Just as the lack of fit model (Heilman, 1983) and role congruity theory (Eagly and Karau, 2002) explain why heterosexual women are less likely to be hired for male-typed jobs because they are perceived to lack the masculine traits presumed to be necessary to be successful in those jobs, these theories also predict the same bias toward gay male applicants. That is, the stereotype of gay men as feminine should lead to the gay male applicant being viewed as a better fit for the female-typed job and more likely to be hired for the female-typed job than the male-typed job. Based on the foregoing, we expect that:

*Hypothesis 1: Applicant sexual orientation will interact with job-gender type to predict respect ratings, such that the heterosexual male will be rated lower on respect when applying for the female-typed job than when applying for the male-typed job, but the gay male will be rated similarly across job gender-types*.

*Hypothesis 2: Applicant sexual orientation will interact with job-gender type to predict ineffectuality ratings, such that the heterosexual male will be rated higher on ineffectuality when applying for the female-typed job than when applying for the male-typed job, but the gay male will be rated similarly across job gender-types*.

*Hypothesis 3: Applicant sexual orientation will interact with job-gender type to predict hiring decisions, such that the heterosexual male will be less likely to be hired when applying for the female-typed job than when applying for the male-typed job, but the gay male will be less likely to be hired for the male-typed job than for the female-typed job*.

## Materials and methods

We carried out an experiment that followed a 2 (gay male, heterosexual male) × 2 (female-typed job or male-typed job) design. The female-typed job was an esthetician and the male-typed job was an auto mechanic. We designed a between-subjects experiment because it allowed us to test causation while also avoiding demand and carryover effects. Further, between-subject designs are more conservative tests than within-person designs (Charness et al., 2012), thus instilling greater confidence in our findings.

### Participants

We recruited employed adults in the United States with experience in hiring (recruitment and selection) through Qualtrics. Participants were randomly assigned to one of the 4 conditions, with approximately equal numbers of male and female participants assigned to each condition. The total number of participants was 128 (female = 66, male = 62). Cell sizes ranged from 30 to 36 participants per condition. For statistical tests used to detect differences, like the ANOVAs performed herein, 30 participants per cell is recommended to achieve sufficient power (Cohen, 1988; Wilson VanVoorhis and Morgan, 2007). The ages of the participants ranged from 19 to 73 years with a mean of 38 years. In terms of sexual orientation, 91% were heterosexual and 9% were gay or lesbian. The highest level of education attained was distributed as follows: high school diploma = 19.5%, some post-secondary = 18%, undergraduate degree = 33.6%, and graduate degree = 28.9%.

### Procedure

Participants who consented to participate in our study were asked to imagine they were the manager at either a spa or garage and were reviewing applicants to fill a full-time esthetician or auto mechanic position. To reinforce the gender-type of the position a list of names of six applicants was supplied. In the esthetician condition, the other five names were female names while in the mechanic condition, they were male names. The participants were instructed that they were currently examining the application of one of these six applicants.

Each participant then read a job description for the position to be filled followed by a background summary for the applicant they were reviewing and then rated the applicant on a series of measures. All background summaries were identical across conditions, with only the applicant's sexual orientation varying. We manipulated sexual orientation by referencing either the applicant's girlfriend or boyfriend. Job descriptions and background summaries are reproduced in Appendix A.

### Measures

Participants rated the experimental applicant on several measures and completed some demographic questions, detailed below. To measure ineffectuality, respect, and likability, we employed items used by Heilman and Wallen (2010).

#### Ineffectuality

Ineffectuality was measured with five items rated on 9-point bipolar scales (Heilman and Wallen, 2010): wimpy, wishy-washy, insecure, spineless, and weak (α = 0.91).

#### Respect

How respect-worthy the participants perceived the stimulus person to be was assessed with one item: “How much do you think Philip is someone who commands respect?” rated on a 9-point scale from completely not respected (1) to completely respected (9) (Heilman and Wallen, 2010).

#### Likability

Likability was assessed with two items. The first item was “How much do you think you would like Philip”? (Heilman and Wallen, 2010). The second asked participants to rate how likable they perceived the applicant to be. Both items were rated on a 9-point scale from completely dislike (1) to completely like (9). Cronbach's alpha was 0.81.

#### Hire

Participants indicated whether they would hire the applicant for the position on a five-point scale from definitely not (1) to definitely (5).

#### Demographics and controls

We asked participants to report their gender (male, female, or other), sexual orientation (gay/lesbian, straight, or other), age, and highest level of education attained. As a potential control variable, we included Morrison and Morrison's (2008) modern prejudice toward gay men and lesbian women scale. It consists of 12 items measured on a 5-point scale from strongly disagree (1) to strongly agree (5). A sample item is: “Many gay men/lesbians use their sexual orientation so that they can obtain privileges.” Higher scores represent more negative attitudes toward gay men and lesbians. For this scale, α = 0.91.

#### Manipulation checks

We included checks of our manipulations of job-gender type and sexual orientation. Participants reported whether the job (esthetician or auto mechanic) was a job most commonly held by only male (1), mostly males (2), both males and females (3), mostly females (4), or only females (5). The higher the score, the more female-typed the job. The lower the score the more male-typed the job. Participants also reported whether the applicant was heterosexual (1), homosexual (2), or the participant didn't know (3).

## Results

To verify our manipulation of job gender-type we conducted an independent samples *t*-test comparing ratings on the job gender-type item across the two job conditions. The test confirmed that we had successfully manipulated job gender-type [*t*_(126)_ = 12.595, *p* = 0.000]. Examination of the means confirmed that participants perceived the job of esthetician to be female-typed (mean = 3.47) and the job of mechanic to be male-typed (mean = 2.10). To our sexual orientation manipulation check question, participants responded either heterosexual, gay, or “I don't know.” Examination of the distribution of responses and as well as the results of chi square tests of the distribution in each condition verified confirmed that we had successfully manipulated the sexual orientation of the applicants (gay condition: *X*^2^ = 37.84, *p* = 0.000; heterosexual condition: *X*^2^ = 59.59, *p* = 0.000).

To ensure differences in perceived applicant likability did not influence ratings on the dependent variables, we performed a one-way ANOVA examining likability ratings across all four (4) conditions. The ANOVA was not significant (*p* > 0.05). We also performed an independent samples *t*-test comparing likability ratings across the two (2) applicant conditions and this test was also nonsignificant (*p* > 0.05).

As recommended by Biskin (1980) and Huberty and Morris (1989), we tested our hypotheses by performing multiple univariate analysis of variance (ANOVAs), rather than one multivariate analysis of variance (MANOVA) because our dependent variables were conceptually independent.

Means on the dependent variables by job and applicant are reported in Table 1. Correlations, overall means, and standard deviations for study variables appear in Table 2.

**Table 1:** Means and standard deviations by condition.

		**Applicant**	
**Outcome**	**Job**	**Gay male**	**Heterosexual male**
Respect^*^	Esthetician	6.97 (1.16)	6.11 (1.77)
	Mechanic	6.65 (1.84)	7.52 (1.36)
Ineffectuality^**^	Esthetician	2.79 (1.35)	3.67 (1.77)
	Mechanic	3.28 (1.67)	2.35 (1.55)
Hire^***^	Esthetician	3.93 (0.58)	3.56 (0.91)
	Mechanic	3.97 (0.84)	4.16 (0.82)

* *Rated on a 9-point scale with larger means representing higher levels of respect*.

** *Rated on a 9-point scale with smaller means representing higher levels of ineffectuality*.

*** *Rated on a 5-point scale with larger means representing higher levels of hiring recommendations*.

**Table 2:** Means, standard deviations, and zero-order correlations.

	**M**	**SD**	**1**	**2**	**3**	**4**	**5**	**6**	**7**	**8**
Applicant sexual orientation^τ^	–	–	1							
Job gender-type^ττ^	–	–	−0.05	1						
Respect	6.78	1.28	−0.01	0.18^*^	1					
Ineffectuality	3.05	1.66	0.01	−0.14	−0.52^**^	1				
Hire	3.89	0.82	−0.07	0.21^*^	0.61^**^	−0.47^**^	1			
Participant gender^τττ^	–	–	−0.05	0.06	0.08	0.08	0.05	1		
Participant sexual orientation^ττττ^	–	–	−0.01	0.02	0.04	−0.04	−0.04	−0.21^*^	1	
Participant age	38.21	12.06	0.00	0.02	0.00	−0.08	−0.04	0.15	−0.13	1

* *Correlation is significant at p < 0.05 (2-tailed)*.

** *Correlation is significant at p < 0.01 (2-tailed)*.

τ *Gay = 1, heterosexual = 2*.

ττ *Female-typed = 1, male-typed = 2*.

τττ *Male = 1, female = 2*.

ττττ *Gay/lesbian = 1, heterosexual = 2*.

**Figure 1:**
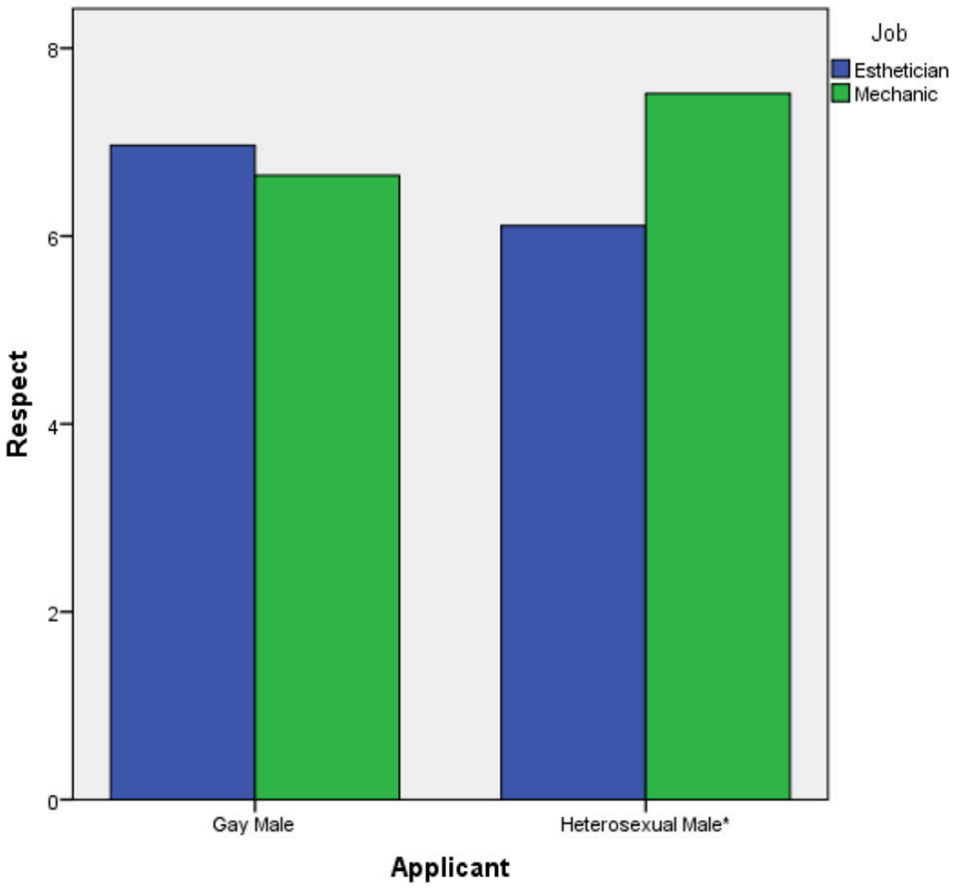
The 2-way interaction of applicant sexual orientation and job gender-type on respect ratings. ^*^Difference at *p* = 0.001.

Because modern prejudice toward gay men was not significantly correlated with any of our study variables (*p* > 0.05) we did not include it as a covariate in our analyses. Hypothesis 1 predicts a 2-way interaction between applicant sexual orientation and job gender-type in the prediction of respect ratings. To test this hypothesis, we conducted a 2 (applicant sexual orientation) × 2 (job gender-type) ANOVA test. In support of hypothesis 1, the 2-way interaction was statistically significant, *F*_(1, 124)_ = 9.635, *p* = 0.002, partial eta^2^ = 0.07. This interaction is displayed in Figure 1. Examination of the figure reveals that the largest different in respect ratings across jobs is in the heterosexual male condition. To further examine this 2-way interaction result, we conducted simple *t*-tests to compare respect ratings across job gender-types for each applicant sexual orientation, employing a Bonferonni corrected alpha of α < 0.025 for each test to maintain a family-wise error rate of α < 0.05. The only statistically significant result was for the heterosexual male, *t*_(65)_ = −3.596, *p* = 0.001, eta^2^ = 0.166. Examination of the means (Table 1) indicated that the heterosexual male applicant received higher respect ratings in the mechanic condition than in the esthetician condition. For the gay male applicant, the difference between respect ratings across jobs was not statistically significant (*p* < 0.025). Thus, hypothesis 1 was supported.

**Figure 2:**
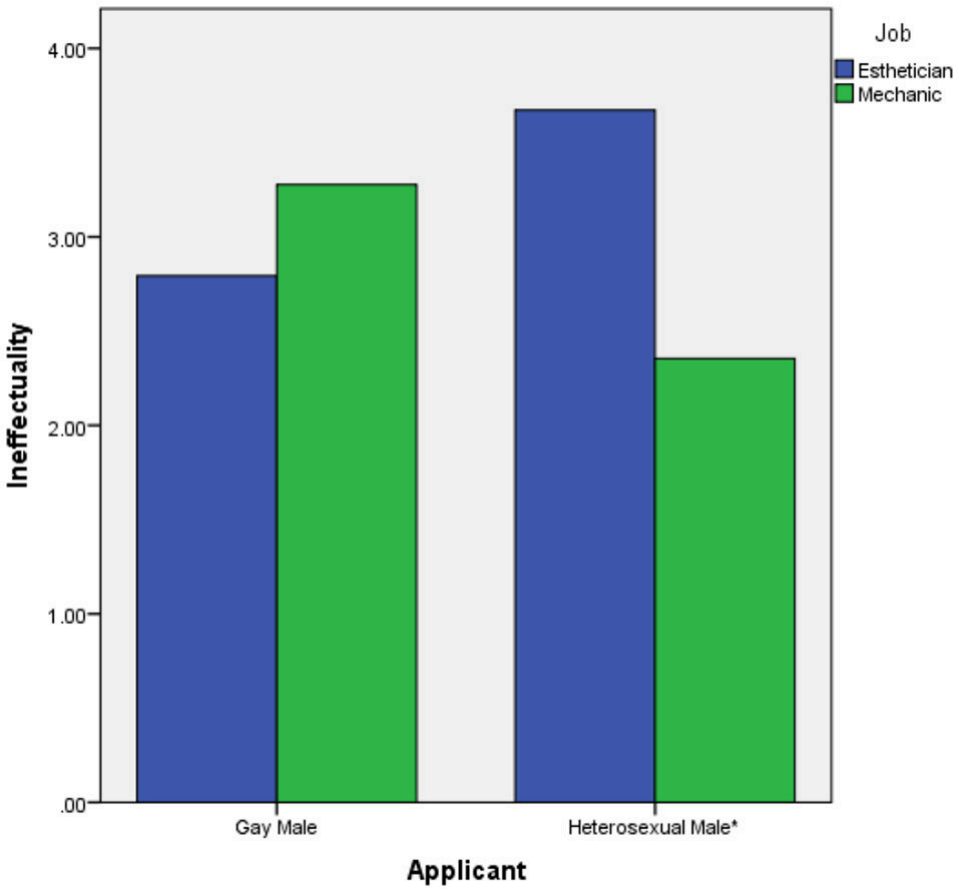
The 2-way interaction of applicant sexual orientation and job gender-type on ineffectuality ratings. ^*^Difference at *p* = 0.001.

To test our second hypothesis, which predicted a 2-way interaction of applicant sexual orientation and job gender-type in the prediction of ineffectuality ratings, we performed a 2 (applicant sexual orientation) × 2 (job gender-type) ANOVA with ineffectuality ratings as the dependent variable. The 2-way interaction was significant, *F*_(1, 124)_ = 10.063, *p* = 0.002, partial eta^2^ = 0.075. This interaction is displayed in Figure 2.

To further probe the 2-way interaction, we performed two simple *t*-tests to compare ineffectuality ratings across job-type for each applicant (α = 0.05). The result for the heterosexual male condition was significant, *t*_(65)_ = 3.216, *p* = 0.001, eta^2^ = 0.137. Examination of the means (Table 1) reveals that the heterosexual male was rated higher on ineffectuality in the esthetician condition than in the mechanic condition. The *t*-test for the homosexual male condition, however, was not significant (*p* > 0.025). Hypothesis 2 was therefore supported.

**Figure 3:**
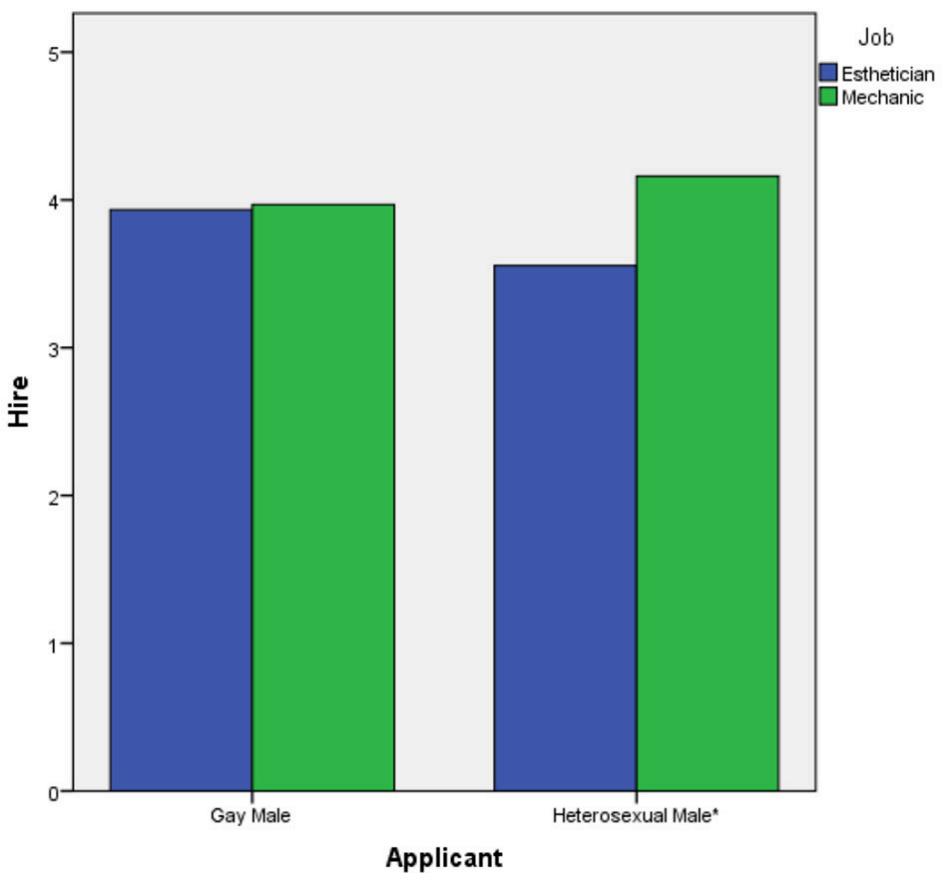
The 2-way interaction of applicant sexual orientation and job gender-type on hire ratings. ^*^Difference at *p* = 0.003.

Our third and final hypothesis predicted a 2-way interaction between applicant sexual orientation and job gender-type in the prediction of hire ratings. To test this hypothesis, we conducted a 2 (applicant sexual orientation) × 2 (job gender-type) ANOVA test. The 2-way interaction was statistically significant, *F*_(1, 124)_ = 4.135, *p* = 0.044, partial eta^2^ = 0.032. This interaction is displayed in Figure 3. Examination of the figure reveals that the largest difference in “willingness to hire” ratings across jobs was in the heterosexual male condition. To further examine this 2-way interaction, we conducted simple *t*-tests to compare hire ratings across job gender-types for each applicant, employing a Bonferonni corrected alpha of α < 0.025 for each test. The only statistically significant result was for the heterosexual male, *t*_(65)_ = −2.905, *p* = 0.003, eta^2^ = 0.115. Examination of the means (Table 1) indicated that the heterosexual male received higher “willingness to hire” ratings in the mechanic condition than in the esthetician condition. For the gay male, the difference between hire ratings across jobs was not statistically significant (*p* > 0.025). Therefore, hypothesis 3 was partially supported.

## Discussion

Based on theory and previous findings with heterosexual targets, we investigated the interaction of gender and sexual orientation stereotypes in influencing the perceptions of male applicants for gender-typed jobs. Our findings showed that participants rated the heterosexual male applicant significantly more ineffectual in the esthetician condition than in the mechanic condition, and rated the heterosexual male more respect-worthy and hirable in the mechanic condition than the esthetician condition. For the gay male applicant, the difference between respect ratings and ineffectuality ratings across jobs was not statistically significant. There was also no significantly different willingness to hire the gay male across the female- and male-typed jobs in our study.

The results regarding heterosexual males are consistent with predictions made by role congruity theory (Eagly and Karau, 2002) and the lack of fit model (Heilman, 1983). The results are also supportive of prior experimental work that did not explicitly divulge the sexual orientation of the job applicant (Heilman and Wallen, 2010). One important contribution this study makes it to extend this work by explicitly incorporating sexual orientation into the study of how gender stereotypes influence evaluations of gay men applying for gender- congruent and -incongruent roles.

Theory and prior research on gender-typed work suggests that we can imply how masculine and/or feminine the participants perceive the applicant to be by how they evaluate that individual when engaged in a gender-typed occupation (e.g., Heilman et al., 2004). Our findings that the gay male's ratings on ineffectuality and respect did not vary across job gender-types is not consistent with the gender stereotypes of gay males being feminine and more similar to heterosexual women than to heterosexual men (Blashill and Powlishta, 2009). The finding that the gay male was viewed as equally hirable for the male-typed job and the female-typed job is also inconsistent with this stereotype or implicit inversion theory (Kite and Deaux, 1987). These results may in fact suggest that the gender stereotypes of gay men are changing.

Two recent studies also support a changing perception. One study, examining the content of gender stereotypes, failed to find support for implicit inversion theory (Clarke and Arnold, 2017). Using an explicit measure of gender stereotypes, Clarke and Arnold (2017) found that perceptions of heterosexual men and women were consistent with prior research, however, gender stereotypes of gay men and lesbians were not inverted. There were no significant differences between the masculinity and femininity ratings assigned to lesbians and those assigned to gay males. The authors proposed that with legal and societal change, the conceptualizations of the typical gay man and typical lesbian were changing. Another study, completed in Germany, found that, when applying for leadership positions, lesbians and gay men were perceived to have equal levels of social skills, a female trait, and competency, a male trait (Niedlich and Steffens, 2015), again suggesting perception of the applicants as androgynous.

Viewed in tandem with these studies, our results may suggest that participants used an androgynous gender stereotype of gay men when making judgments of suitability for employment. The gay male's respect and ineffectuality ratings did not vary by job gender-type and he was equally likely to be respected, considered effectual, and hired in both job conditions (mechanic and esthetician). In other words, the results are consistent with Clarke and Arnold's (2017) finding that gay men were perceived as possessing both stereotypically masculine and feminine attributes. It may be that the gay male applicant was perceived as androgynous, and although further corroborating studies explicitly measuring the perceived androgyny of gay males are necessary, it may be that implicit inversion theory is in need of revision.

When hiring decisions in a gender-typed job are being made, and when the sexual orientation of the applicant is known or implied, our findings suggest that gay men would be perceived as equally suitable in both traditionally male and female jobs. Given mixed findings in the nascent work in this regard, we suggest that future studies are required to determine whether implicit inversion theory requires revision. Has there been an actual change in how gay men are perceived in terms of masculinity and femininity given societal changes that have been unfolding, or are our results an artifact of our data or method? This question can only be answered with further research and theorizing.

Notwithstanding that a field study would be superior with respect to generalizing to the real employment setting, we attempted to increase the ecological validity of our study by recruiting participants with actual hiring experience instead of undergraduate students. In this regard some of the findings that differ from past work on gender stereotypes and perceptions of job fit may be a result of differences in the demographics of our sample. A second limitation is that we did not explicitly measure gender stereotypes in this study. While we have based the explanation of our findings on this theory, future work could incorporate explicit measures of gender stereotypes (e.g., communality—Heilman and Okimoto, 2007) and test its explanatory power. Third, future work should incorporate a broader range of jobs, including gender-neutral jobs and jobs that are perceived as equal in respect and status. In our study, the male-typed job, auto mechanic, was viewed with greater respect than the female-typed job of esthetician. The 2-way interaction of job-gender type and applicant sexual orientation was still significant when forced to compete with this main effect in our analysis, however, future work should compare applicant ratings across jobs that are viewed as being of equal status and equally respect-worthy as well as those that are more subtly gender-typed. Further, it is also possible that our participants viewed the job of esthetician as a “gay job” (Anteby and Anderson, 2014). Research is therefore necessary to determine whether a gay male applicant would be evaluated more positively than heterosexual male for a female-typed job that is not characterized as a “gay job,” such as nurse or administrative assistant.

In addition to directions mentioned above, future research could extend this work by focusing on how gender stereotypes impact the perceptions of effectuality, respect, and willingness to hire lesbians, as well as evaluations of lesbians when employed in gender-typed jobs. It would be interesting to identify whether descriptive gender stereotypes of lesbians are also changing. Future experimental studies would also provide insight into these processes through manipulating various aspects of gender. Just as previous research in this area has used the categories of male and female and assumed homogeneity within these categories, the current study relies on the assumption that all heterosexual men are similar and all gay men are similar. While this is still an informative approach as stereotypes are by their very nature over-generalizations based on group membership, experimental studies could manipulate individuating information along the lines of masculinity and femininity, in order to disentangle these effects. For example, one could present a gay male who occupies a female-typed job but who possesses highly masculine personality traits. Increasing the amount of the information provided about the stimulus person will not only help to fine-tune theory, but may also indicate whether gender stereotypes are less relied upon when more individuating information is provided as has been proposed in past work (Powell, 1993). Further, there is value in future studies explicitly measuring the masculinity and femininity of the job applicants being rated. In this way, the androgyny of the gay male applicant could be measured rather than merely inferred.

At a macro-level our results may suggest that society is becoming more accepting of gay men, and this acceptance is at the heart of why we found no differences for the gay applicant with regards to hiring decisions. Indeed, with an increasing focus on diversity in the workforce, individuals with recruiting experience may be more acutely aware than others of the inequity experienced by gay men, and they may have purposefully engaged in conscious deliberation to come to decisions that were equitable for gay men in this experiment. Perhaps this level of deliberation did not apply in the case of the heterosexual male, as this group may not typically be viewed as a group that has suffered discrimination in the past. This conjecture also suggests that ethical theory may be a useful lens through which to view our findings. For example, justice theory (e.g., Colquitt, 2001) would suggest that hiring is a distributive outcome, and those with hiring experience may be attuned to the moral imperative to equate outcomes for groups that have been disadvantaged in the past. As well, this may also relate to the argument that it is important to approach organizational diversity efforts from a moral/ethical perspective, not just a business case perspective (e.g., Noon, 2007; Gotsis and Kortezi, 2013). Future research could investigate whether these possible explanations for our findings hold up to empirical scrutiny.

Research on gender-inconsistent employment calls attention to backlash and discrimination experienced by individuals engaged in such work. These phenomena, according to role congruity and lack of fit theories, occur, at least in part, through the operation of gender stereotypes. Past research on sexual orientation has shown that the gender stereotypes of gay and lesbian individuals tend to be inverted; that is in the opposite direction of those about heterosexual individuals. However, the current study found little difference in how gay men were perceived when applying for female-typed vs. male-typed jobs. These findings may suggest that gender stereotypes of gay men are changing and implicit inversion theory may no longer apply.

## Ethics statement

This study was carried out in accordance with the recommendations of the Interdisciplinary Committee on Ethics in Human Research, Memorial University of Newfoundland, with written informed consent from all subjects. All subjects gave written informed consent in accordance with the Declaration of Helsinki. The protocol was approved by the Interdisciplinary Committee on Ethics in Human Research, Memorial University of Newfoundland.

## Author contributions

All authors listed have made a substantial, direct and intellectual contribution to the work, and approved it for publication.

### Conflict of interest statement

The authors declare that the research was conducted in the absence of any commercial or financial relationships that could be construed as a potential conflict of interest.

## Appendix A

### Instructions

Imagine that you are the manager of ABC Spa and Salon. Currently the spa is seeking to fill a full-time esthetician position. Six individuals have applied for the position. Their first names are listed below:

List of Applicants:

1. Juanita
2. Megan
3. Victoria
4. Emily
5. Philip
6. Donna

You are currently examining Philip's application. To make your evaluation, you will read the job description for the esthetician position and Philip's background summary. You will then be asked to rate Philip on a feedback form.

### ABC SPA and salon job description

#### Position: full-time esthetician

##### General description

This position is for a full-time esthetician.

Primary job responsibilities:

1. Provide a full range of esthetic services to clients including facials, nail art, and design, aromatherapy, pedicures, make-up application, hair removal, and body wraps and treatments.
2. Stay up to date on the latest makeup trends and esthetic techniques.
3. Recommend and promote the spa's product line, including make-up, and skin care products.

#### Instructions

Imagine that you are the manager of ABC Auto Garage. Currently the garage is seeking to fill a full-time auto mechanic position. Six individuals have applied for the position. Their first names are listed below:

List of Applicants:

1. Juan
2. Michael
3. Victor
4. Steve
5. Philip
6. David

You are currently examining Philip's application. To make your evaluation, you will read the job description for the auto mechanic position and Philip's background summary. You will then be asked to rate Philip on a feedback form.

### ABC auto garage job description

#### Position: full-time auto mechanic

##### General description

This position is for a full-time automotive mechanic.

Primary job responsibilities:

1. Provide a full range of maintenance and repair services including work on engine systems, drive lines, electrical, steering, braking systems, and body components.
2. Stay up to date on the latest automotive technology and advances.
3. Recommend and promote the garage's full range of services, including maintenance and repair.

#### Candidate's background summary (esthetician condition)

##### Philip

Philip was born in a town about an hour away from ABC Spa and Salon. He graduated from the Esthetics Program at ABC Community College about ten years ago. He then returned to his hometown with his boyfriend (girlfriend), Gary (Sarah), to work as an esthetician. Five years ago Philip and Gary (Sarah) relocated to this area and Philip took a job at a local spa. Since then he has been working part-time as esthetician and is now seeking a full-time position. Philip enjoys being an esthetician and regularly attends training to stay current in his field.

#### Candidate's background summary (mechanic condition)

##### Philip

Philip was born in a town about an hour away from ABC Auto Garage. He graduated from the Automotive Service Technician Program at ABC Community College about ten years ago. He then returned to his hometown with his girlfriend (boyfriend), Sarah (Gary), to work as an auto mechanic. Five years ago Philip and Sarah (Gary) relocated to this area and Philip took a job at a local garage. Since then he has been working part-time as a mechanic and is now seeking a full-time position. Philip enjoys being a mechanic and regularly attends training to stay current in his field.
